# Intersystole in Man

**Published:** 1913-10

**Authors:** 


Intersystole In Man, G. Etienne, *Arch des Mal. du Coeur, des Vaisseaux, et du Sang,* 1913, VI, 161. Étienne publishes polygraphic tracings from six persons; three with hypertrophy of the heart and three perfectly normal persons, which show the wave described by Pachon in the dog, by Chauveau in the horse, and by Pezzi and Sabri in man as intersystole. Étienne considers the intersystole as an active phenomenon, and not as a simple elastic reaction of the ventricle secondary to overdistension of the auricle. In man the intersystolic wave (i) appears at the end of the auricular contraction and just before the ventricular contraction; after the auricular muscle has completely returned to the period of rest and before the ventricular muscle begins to contract. It is, graphically, presystolic (that is to say, it occurs in the presphygmic period); physiologically it is a systolic phenomenon, due to the contraction of the papillary muscles. The closure of the auriculoventricular valves, according to Étienne, is composed of three parts: (1) obliteration by concentric pressure upon the walls of the valve leaflets by the blood filling the ventricle at the end of the auricular contraction. (2) Accentuation of this valvular closure and fixation of the closed valves by traction resulting from the contraction of the papillary muscles. (3) Ventricular contraction, completing the occlusion at the beginning of systole by energetic and passive tension of the papillary muscles.

